# Optimize planting density and nitrogen fertilizer rate on green cob and grain yield of maize (*Zea mays* L.) under irrigation in North Shewa, Ethiopia

**DOI:** 10.1016/j.heliyon.2024.e36367

**Published:** 2024-08-18

**Authors:** Degu Temeche, Elias Getachew, Getachew Hailu

**Affiliations:** Amhara Regional Agricultural Research Institute, Debre Birhane Agricultural Research Center, P.o.box 112, Debre Birhan, Ethiopia

**Keywords:** Plant density, Nitrogen rate, Grain yield, Green cob

## Abstract

Nowadays producing maize for green cob is a profitable business in addition to producing maize for grain yield. High plant density has been widely used to enhance grain yield in maize. A field experiment was conducted at four locations for two consecutive years (2021–2022) to determine the optimum plant density and nitrogen level and to assess the advantage of selling the green cob rather than the grain yield. The experiment was laid out in a Randomized Complete Block Design in a factorial arrangement with three replications. Three plant populations (44,444, 66,666, and 88,888 plants ha^−1^) with five nitrogen levels (0, 46, 92, 138, and 184 kg ha^−1^ were combined by factorial combinations and tested in the experimental plots to select the optimum level for high grain and green cob yield. The maximum grain yield (8656 kg ha^−1^) was obtained under the highest plant population (88,888 plants ha^−1^), and from tested nitrogen rates 46, 92, and 138 kg nitrogen ha^−1^ gave statically similar higher yield. Based on partial budget analysis, the application of 92 kg ha-1 nitrogen under 88,888 plant population was more economically feasible than other treatment combinations. This result showed 23.42 % yield advantages compared to the positive control. In addition, applying 138 kg N under 88,888 plant densities was the most profitable compared to other combinations to produce green cob. Thus, the blanket recommended plant population, 44,444 plants ha^−1^ with application of 46 kg N ha^−1^ is insufficient for maize green cob and grain yield production. Rather use of 88,888 plants ha^−1^ with the application of 92 kg N ha^−1^ for grain yield production and the use of 88,888 plants per ha^−1^ with the application of 138 kg N ha^−1^ is profitable for the production of maize green cob.

## 1 Introduction

Maize (*Zea mays*) is one of the most important cereal grain crops used as the human diet, livestock feed, and raw material for various industries in large parts of the world [1]. The national average productivity of maize is about 41.95 qt ha^−1^, in the Amhara region 42.86 qt ha^−1,^ and in the North Shewa zone 36.00 qt ha^−1^ [2], while that of other countries is up to 62 qt ha^−1^. This indicates that the maize productivity of North Shewa farmers is extremely far below the world's average even the national and regional average yield due to several biotic and abiotic factors. Among many factors, declining soil fertility, poor agronomic practice, limited use of agricultural input, insufficient technology generation, and poor seed quality particularly affect Ethiopian maize productivity considerably [3].

Recent increases in maize grain yield can be attributed to genetic advances and improved agronomic practices, including optimizing plant populations [4]. Plant population has a strong influence on maize grain yield [5], but this relationship is highly variable [6] and can be affected by factors such as rainfall, tillage system, fertilization, and soil type.

The study area of Kewet and Efratanagidem district is dominated by a midland agroecological setting where maize is planted in January/February and harvested as green maize as well as grain yield under irrigation. Every year farmers in the study area sell out green maize at low prices due to the size of the cob. The cob length and diameter are highly related to plant density and nitrogen fertilizer. In numerous places along the road green maize is currently being sold in large quantities. A major trading cob for green maize are Debrebirhan, Shewarobit, and Ataye markets. There is a controversy over the question, of whether farmers are profitable by doing this or suffer for extra cost than that of producing grain yield.

Plant population has important effects on vegetative [7] and reproductive development of maize [8,9]. Maize is more sensitive to variations in plant density than other members of the grass family. At low densities, many modern maize hybrids don't tiller effectively and quite often produce only one ear per plant. Therefore, maize does not share the trait of most tillering grasses of compensating for low leaf area and small number of reproductive units by branching [10]. On the other hand, the use of high populations enhances interplant competition for light, water, and nutrients. This may be detrimental to the final yield because it stimulates apical dominance, induces barrenness, and ultimately decreases the number of ears produced per plant and kernels set per ear [11]. For each production system, there is a population that maximizes the utilization of available resources, allowing the expression of maximum attainable grain yield in that environment [12]. Higher plant populations produce 25 % more grain yield and 38 % more biomass as compared with low plant populations [13]. Plant density is related to production environments because when density exceeds the ideal level for a given environment, there is a negative impact per plant, which reduces crop yield [14]. This occurs because the number of plants per area is related to greater efficiency in the use of available resources [15].

Nitrogen is the main limiting nutrient after carbon, hydrogen, and oxygen for the photosynthetic process, phyto-hormonal, proteomic changes, and growth-development of plants to complete its lifecycle. Excessive and inefficient use of N fertilizer results in enhanced crop production costs and atmospheric pollution [16]. Since it is highly mobile, it is subjected to greater loss from the soil-plant system [17]. Even under the best management practices, 30–50 % of applied N is lost through different agencies and, hence,the farmer is compelled to apply more than the actual need for the crop to compensate the loss [17]. The loss of N not only harasses the farmers but also has a hazardous impact on the environment [18]. Higher chemical fertilizer inputs for sustainable crop production cause soil degradation and environmental pollution [8]. Nitrogen fertilization significantly increased grain yields per plant and per unit area for all hybrid maize. The theoretical optimum N application rates for high yield for hybrids released in the 1970s and 1980s were about 280 and 360 kg ha^−1^, and the hybrids from the 1990s and 2000s showed the highest yield at 330 kg ha^−1^ N [19].

There is a great possibility to enhance maize productivity by increasing its planting density with increasing N fertilizer rate [20]. Nowadays, Ethiopian maize producers require more information about what combination of N-fertilizer level and plant density precisely increases maize yield, while the Government is promoting intensive crop production including maize to enhance grain production in the country in general. However, optimum planting density and N fertilizer are not yet well determined in the study area. Therefore, the research was conducted with the objectives (i) to determine the optimum plant population and appropriate level of nitrogen for obtaining a higher yield of maize, and (ii) to assess the advantage of selling green maize over maize grain yield.

## 2 Materials and methods

### 2.1 Description of the study area

**Fig. 1:**
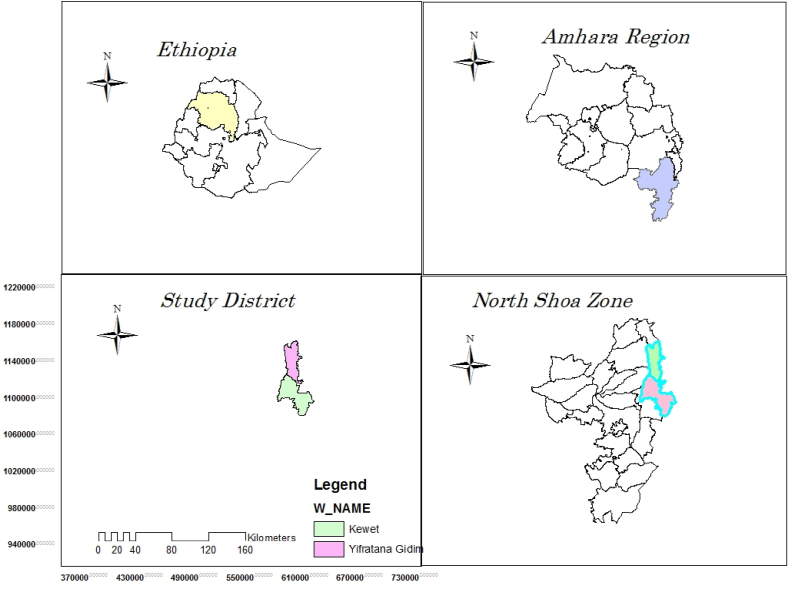
Location map of the study district.

The experiment was conducted on farmers’ fields and on-stations at two districts (Efratanagidem and Kewet) under irrigation growing season for two years (2021 and 2022). The geographical location of the experimental sites are located between 9^0^ 96′ 25″ to 10^0^ 17′ 46″ N latitude and 39^0^ 54′ 10″ to 39^0^ 88′ 29″E, with longitude, and altitude ranging from 1300 to 1543 m.a.s.l. (Fig. 1). The major crops grown in the area are sorghum, onion, tef, and tomato and from livestock cattle and goats are dominant for the area.

### 2.2 Soil analysis

Composite soil samples were taken at 0–30 cm depth using an auger in a zigzag pattern before planting from each experimental site in each experimental year. The soil samples were then analyzed at the Debre Birhan Agricultural Research Center soil laboratory for chemical properties (Soil pH, Organic carbon, Organic matter, Total N, and Available phosphorus) using standard laboratory procedures and classified according to Ref. [21]. Soil water reaction determined by distilled water suspension (Table 1)

**Table 1:** The mean value of some physicochemical properties of the soil of experimental sites.

Soil characteristics	Rating	Rating range	Analysis method
Soil pH	6.95	Neutral	1:2.5 soil-to-water ratio
Organic carbon (%)	1.42	Low	Walkley-Black
Total Nitrogen (%)	0.14	Medium	Kjeldahl
Organic matter (%)	2.44		
Available P (ppm)	28.92	Medium	Olsen
Texture clay			
Sand (%)	27.00		
Clay (%)	52.75		
Silt (%)	20.25		

### 2.3 Treatments and experimental design

Treatments consisted of a complete factorial combination of three plant densities (44,444, 66,666, and 88,888 plants per hectare) with five (0, 46, 92, 138, and 184 N kg ha^−1^) (Table 2). Crop variety BH-546 was used. The treatment setup that comprises 44,444 plants ha^−1^ with the application of 46 kg N ha^−1^ was used as a positive control. The experiment was laid out as a randomized complete block design (RCBD) in a factorial arrangement with three replications. The gross plot size was 3.75m × 3m (11.25 m^2^), but the net plot size of 2.25m × 3m (6.75 m^2^) was used for harvesting to minimize the border effects. From three central rows, one row was used for green cob data and the other two rows for grain yield data. The blocks and plots were separated by 2m and 0.75m wide space respectively. To maintain plant populations 44,444, 66,666, and 88,888 plants ha^−1^, we used plant-to-plant distances of 30 cm, 20 cm, and 15 cm, respectively were maintained.

**Table 2:** Treatment combination.

Treatment no	Treatment combination	Plants ha^−1^	Plant spacing (cm)	Kg Nitrogen ha^−1^
1	D1N1	44,444	75 x 30	0
2	D1N2	44,444	75 x 30	46
3	D1N3	44,444	75 x 30	92
4	D1N4	44,444	75 x 30	138
5	D1N5	44,444	75 x 30	184
6	D2N1	66,666	75 x 20	0
7	D2N2	66,666	75 x 20	46
8	D2N3	66,666	75 x 20	92
9	D2N4	66,666	75 x 20	138
10	D2N5	66,666	75 x 20	184
11	D3N1	88,888	75 x 15	0
12	D3N2	88,888	75 x 15	46
13	D3N3	88,888	75 x 15	92
14	D3N4	88,888	75 x 15	138
15	D3N5	88,888	75 x 15	184

Where: D1 = 44,444, D2 = 66,666, D3 = 88,888 Plants ha^−1^ N1 = 0, N2 = 46, N3 = 92, N4 = 138, N5 = 184 KG Nitrogen fertilizer per hectare.

### 2.4 Agronomic management

Planting time was varied from location to location but all planting times were done between Feb 24 to Mar 5. Planting was done with one additional seed per hole and seedlings were thinned to the required plant per plot two weeks after planting to keep a good stand seedling for each treatment. A full dose of phosphate fertilizer in the form of TSP at the national recommended rate of 60 kg P_2_O_5_ ha^−1^ was applied uniformly to all plots at the time of sowing. Nitrogen was applied as a split application 1/3 at the time of sowing, 1/3 at knee height (5–6 leaf stage), and 1/3 at pre-tasseling stages of maize. All other agronomic practices including weeding, hoeing, field pest control, irrigation, and other practices were done as per research recommendations for maize. Stoke borer was the major problem for the research area, so to control this we used insecticide (karate).

### 2.5 Data collection

Days to 50 % tasseling: it was recorded as the number of days from planting to the date at which 50 % of the plants in a plot produced tassels. Days to 50 % maturity: it was recorded on the date at which 50 % of the cob per plot reached physiological maturity. Data for plant height (cm), number of cobs per plant, cob length (cm), and cob diameter (cm) were recorded from ten randomly taken samples per plot. Above-ground biomass and grain yield (kg ha^−1^) data were taken from the net plot area at harvesting. Plants of the middle two rows on a 4.5 m^2^ area were harvested and weighed. The cobs were then shelled, and dried in the bright sunshine and their moisture content was measured. The grain yield of each plot was then calculated and converted to kg ha^−1^. Data for green cob was recorded from one central row by stratified sampling during the ideal green cob maturity stage by visual observation. Hundred kernels weight (g): it was determined by counting 100 grains and weighing them on a sensitive balance. The weight was adjusted to a 12.5 % moisture level.

### 2.6 Statistical analysis

Data analyses were conducted according to the principles of analysis of variance, using SAS Software version 9.0, [22]. Significant differences among means were determined by the least significant difference (LSD) test at 5 % probability. The homogeneity of variances was tested using the Bartlett test. According to the homogeneity test, all traits were homogenous.

### 2.7 Economic analysis

Economic analysis was done to investigate the economic feasibility of the treatments. The price of maize that farmers received from the sale was calculated based on the current market price of maize for grain yield and the price of green cob for green cob yield. The mean field price was gained by simple assessment of farmers’ prices in the vicinity of the experimental field after harvest (May and June) for green cob and grain yield respectively in both years. The total variable costs including the cost of hybrid seed, cost of fertilizer, and labor were also calculated based on the current price (the cost of fertilizer was obtained Office of Agriculture). The net return was calculated by subtracting the total variable cost from the total return. Total return was calculated with that grain yield (kg ha^−1^) and stalk yield multiplied by field price which is money gained from the sale of the grain and stalk. Finally, to assess the cost and benefit associated with different treatments, the partial budget analysis was done following the method suggested by Ref. [23].

## 3 Results and discussion

The analysis of variance showed that the number of cobs per plant, cob length, biomass yield, and grain yield were significantly affected by the main effect of plant population and nitrogen fertilizer while except days to tasseling other parameters showed non-significant by interaction effect (Table 3).

**Table 3:** Summary of probability values of combined analysis of variance over environments (year*loc), and treatments (plant population * nitrogen rate).

Source	DF	DT	DM	PHT	NCPP	CD	CL	BIO	SY	GY	HI	HSW
Year	1	0.2112	0.5411	<0.0001	<0.0001	<0.0001	<0.0001	<0.0001	<0.0001	<0.0001	<0.0001	0.182
Location	1	0.0005	0.9805	<0.0001	0.0029	<0.0001	<0.0001	<0.0001	<0.0001	<0.0001	<0.0001	<0.0001
Rep(Loc)	4	0.5259	0.436	0.0006	<0.0001	0.0073	0.0234	0.5813	0.3974	0.1412	0.0019	0.0416
Year*Loc	1	0.1362	0.9026	<0.0001	0.0007	<0.0001	<0.0001	<0.0001	<0.0001	<0.0001	0.342	0.0001
Plant population (PP)	2	**0.086**	**0.6992**	**0.0352**	**0.0259**	**<0.0001**	**<0.0001**	**<0.0001**	**<0.0001**	**<0.0001**	**<0.0001**	**0.0011**
Year*PP	2	0.1373	0.0338	0.267	0.9641	0.0004	<0.0001	<0.0001	<0.0001	0.001	0.5886	0.598
Loc*PP	2	0.955	0.971	0.727	0.1798	0.0256	0.3232	0.0004	0.0048	<0.0001	0.0023	0.0623
Year*Loc*PP	2	0.4036	0.573	0.5613	0.0002	<0.0001	0.0001	<0.0001	<0.0001	0.0003	0.9419	0.6881
Nitrogen rate (NR)	4	**0.064**	**<0.0001**	**0.1265**	**0.009**	**0.0568**	**<0.0001**	**<0.0001**	**<0.0001**	**0.003**	**<0.0001**	**0.0618**
Year*NR	4	0.4847	0.6744	0.0165	0.0218	0.0005	<0.0001	<0.0001	<0.0001	<0.0001	0.5616	0.5691
Loc*NR	4	0.1566	0.3192	0.0084	0.4029	0.0464	0.0009	<0.0001	<0.0001	<0.0001	0.2832	0.1185
Year*Loc*NR	4	0.335	0.0358	0.3189	0.8541	0.0091	<0.0001	<0.0001	<0.0001	<0.0001	0.6526	0.1677
PP*NR	8	**0.0003**	**0.1446**	**0.5513**	**0.513**	**0.3877**	**0.685**	**0.1338**	**0.0759**	**0.3263**	**0.3002**	**0.0905**
Year*PP*NR	8	0.6982	0.9714	0.1425	0.3985	0.4949	0.3997	0.9102	0.8587	0.9889	1	0.4209
Loc*PP*NR	8	0.1216	0.0592	0.2311	0.7837	0.7595	0.3056	0.3202	0.282	0.0966	0.0039	0.0024
Year*Loc*PP*NR	8	0.5072	0.373	0.7748	0.8968	0.1342	0.3987	0.611	0.4718	0.876	1	0.0304
Cv		3.31	2.96	8.57	9.27	3.86	5.81	17.16	19.08	16.30	6.77	5.17

Where, DF = Degree of freedom, DT = Days to tasseling, DM = Days to maturity, PHT=Plant height, NCPP=Number of cob per plant, CD=Cob diameter, CL=Cob length, BIO=Biomass, SY=Straw yield, GY = Grain yield, HI=Harvest index, HSW=Hundred seed weight, and CV = coefficient of variation.

### 3.1 Phenological and growth parameters of maize

The analysis result showed that there was no significant difference in days to tasseling while days to maturity and plant height showed significant differences due to applied nitrogen rate and population density respectively (Table 3) nitrogen rates 138 and 184 kg ha^−1^ statically similar data were given delayed maturity as compared to other nitrogen rates. The maximum days to 50 % maturity (105.97 days) was recorded from the maximum nitrogen rate of 184 kg ha^−1^ (Table 4).

**Table 4:** Main effect of population and Nitrogen rate on growth, yield-related parameters, and productivity of maize over year and location.

Population density	Days to tasseling	Days to maturity	Plant height (cm)	Number of cobs per plant	Cob diameter (cm)	Cob length (cm)
44,444	55.92	102.68	211.82^b^	1.14^a^	15.57^a^	19.57^a^
66,666	55.28	102.97	220.61^a^	1.09^b^	15.18^b^	18.53^b^
88,888	55.25	102.5	217.40^ab^	1.10^b^	15.07^b^	18.09^c^
LSD	NS	NS	6.7156	0.0373	0.2132	0.3935
Nitrogen rate						
0	54.86	100.11^c^	209.6	1.08^b^	15.21	17.67^c^
46	55.42	100.58^bc^	220.01	1.09^b^	15.08	18.13^c^
92	55.5	101.92^b^	216.25	1.09^b^	15.3	18.75^b^
138	56.17	105.00^a^	219.52	1.14^a^	15.29	19.24^b^
184	55.47	105.97^a^	217.68	1.15^a^	15.49	19.85^a^
LSD	NS	1.42	NS	0.05	NS	0.51

Values within a column followed by the same letter are not significantly different at a 5 % probability level. LSD = Least significant difference; NS = None significant.

The shortest plants were obtained under 44,444 plants per hectare while the other two densities were given statically similar highest plant height (Table 4). The plant height increased with an increase in plant density. The increase in the plant height at the highest plant population may be due to strong competition among the plants for sunlight.

Cob length like that of the plant height, the combined analysis of variance revealed that it a highly significant. The cob length increased with an increase in the nitrogen rate. The increase in the cob length with an increase in the rate of nitrogen application could be attributed to the positive effect of N on cob growth due to more availability of N throughout the growing period.

The number of cobs per plant, cob diameter, and cob length showed significant differences in population density the lower population gave the highest value for the listed parameters (Table 4). On the other hand, the higher number of cob and cob length were observed from the highest two nitrogen rates (138 kg ha^−1^ and 184 kg ha^−1^) statically similar) (Table 4).

### 3.2 Above ground biomass yield

The combined analysis of variance showed a significant effect due to the main effect of plant population density and nitrogen fertilizer rate. But the interaction showed non-significant (Table 3). Maximum biomass (25644.1 kg ha^−1^) was recorded under the highest plant density (88,888 plants per hectare) while from applied nitrogen rate, the highest rate (184 kg ha^−1^ N) were gave the lowest biomass yield this could be stalk logging effect. Even if the data was not enough for analysis there was stalk lodging at a higher nitrogen rate (up to 20 % lodging was observed visually). The rest nitrogen rates were gave statically similar higher biomass yield (Table 5).

**Table 5:** Main effect of population and Nitrogen rate on growth, yield-related parameters, and productivity of maize over year and location.

Population density	Biomass (kg ha^−1^)	Stover yield (kg ha^−1^)	Grain yield (kg ha^−1^)	Harvest index (%)	Hundred seed weight (gm)
44,444	15728.4^c^	9011.7^c^	6769c	43.83^a^	31.57^a^
66,666	20475.2^b^	12732.9^b^	7869^b^	39.40^b^	31.37^a^
88,888	25644.1^a^	17091.5^a^	8656^a^	34.43^c^	30.52^b^
LSD	1279.2	893.07	457.59	0.9604	0.5822
Nitrogen rate kg ha^−1^					
0	20521.1^a^	13359.3^ab^	7161.8^c^	36.33^c^	31.83
46	21886.4^a^	13871.9^ab^	8014.4^ab^	38.14^b^	31.50
92	22138.4^a^	14311.5^a^	8297^a^	38.92^b^	31.03
138	20768.8^a^	12964.7^b^	7804.1^ab^	39.19^b^	30.31
184	17764.9^b^	10219.5^c^	7545.3^bc^	43.53^a^	31.04
LSD	1651.40	1152.90	590.75	1.24	NS

Values within a column followed by the same letter are not significantly different at a 5 % probability level. LSD = Least significant difference; NS = None significant.

### 3.3 Grain yield

The combined results of the analysis showed that highly significant (P < 0.01) grain yield difference for the plant population and significant at (p < 0.05) for the applied nitrogen fertilizer rate while the interaction were showed non-significant (Table 3). The grain yield of maize increased with increasing plant density. Maximum grain yield (8656 kg ha^−1^) was obtained at the plant density of 88,888 plants ha^−1^. Similarly, Grain yield was also increased with increasing nitrogen rate up to optimum. From the tested nitrogen rate the maximum grain yield (8297 kg ha^−1^) was obtained from the rate of 92 kg ha^−1^ nitrogen, but statically similar grain yield produced from 46 to 138 kg ha^−1^ nitrogen, while the maximum nitrogen rate (184 kg ha^−1^) gave lower yield than other rates (Table 5). Treatments that received maximum nitrogen showed up to 20 % lodging that is why the yield of the maximum nitrogen declined. The lowest grain yield was observed from the lowest population 44,444 plants per hectare and from no fertilizer applied (0 rate of nitrogen) 6769 kg ha^−1^ and 7161.8 kg ha^−1^ respectively (Table 5). Compared to the standard control of 44,444 plants ha^−1^ with the application of 46 kg N ha^−1^, the mean grain yield was increased by 24 % when the maize crop was sown at 88,888 plants ha^−1^ with the application of 92 kg N ha^−1^ (Table 5). In general, the grain yield ha^−1^ was increased with the increase in plant density and nitrogen rate, although, economically feasible grain yield was achieved under 88,888 plants per hectare with the application of 92 kg N ha^−1^. The increase in maize grain yield under high plant density might be due to the efficient utilization of available resources like nutrients, water, air, and solar radiation. Farnia [24] reported that plant shortage per unit area prevents maximum usage of production parameters while over-density can increase the competition and decrease the yield.

### 3.4 Economics

**Fig. 2:**
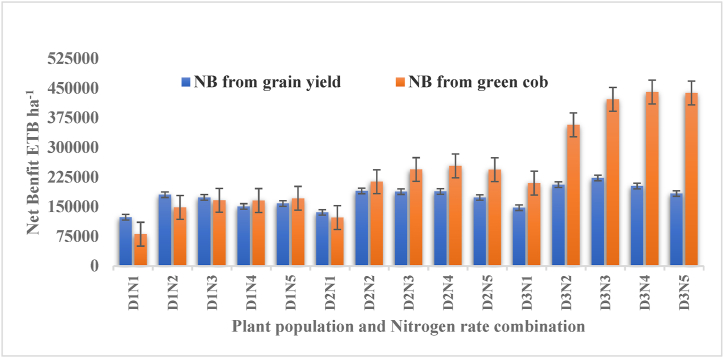
Interaction effect on economic benefit of both maize grain yield and green cob.

Economic analysis was performed to investigate the economic feasibility of the treatments. The price of maize that farmers received from the sale was calculated based on the current market price of maize for grain yield and the price of green cob for green cob yield. The total variable costs including the cost of hybrid seed, fertilizers, and labor were also calculated based on the current price. The net return was calculated by subtracting the total variable cost from the total return. Total return was calculated with that grain yield (kg ha^−1^) and stalk yield multiplied by field price which is money gained from the sale of the grain and stalk. Finally, to assess the cost and benefit associated with different treatments, the partial budget analysis was done following the method suggested by Ref. [23]. The mean field price was obtained by simple assessment of farmers’ prices in the vicinity of the experimental field after harvest (May and June) for green cob and grain yield respectively in both years. The highest net benefit (Birr 222,726) was obtained with a plant population of 88,888 plants ha^−1^ with the application of 92 kg N ha^−1^ in producing maize as a grain (Table 7). Maize production as green cob the highest net benefit (440, 571.70 ETB) was obtained with a plant population of 88,888 plants ha^−1^ with the application of 138 kg ha^−1^ (Table 6). The economic analysis indicated that the higher plant population is basic for maize production as grain and green cob (Fig. 2).

**Table 6:** Dominance and marginal rate of return analysis for the effect of population and nitrogen rate on green cob of maize, combined over years (2021 and 2022).

Density in kg ha^−1^	N rate kg ha^−1^	Bio t ha^−1^	GCNP	Adjusted GCNP	GBN ETB ha^−1^	TVC ETB ha^−1^	NB ETB ha^−1^	MC	MNB	MRR%
27.56	0	12.33	32249.9	29024.9	80535.8	0.0	80535.8			
41.33	0	11.89	48749.3	43874.3	121405.5	931.0	120474.6	931.0	39938.8	4290.0
55.11	0	11.57	65499.2	58949.3	171559.9	1862.3	169697.5	931.4	49223.0	5285.1
27.56	46	17.42	44666.5	40199.9	152744.6	4600.1	148144.6	2737.7	D	
41.33	46	15.84	63499.0	57149.1	218956.0	5531.0	213425.0	931.0	43727.5	4697.0
55.11	46	15.69	87999.0	79199.1	363866.4	6462.4	357404.0	931.4	143979.0	15459.1
27.56	92	17.56	45333.2	40799.9	175083.3	8800.1	166283.2	2337.7	D	
41.33	92	16.92	64499.0	58049.1	253920.6	9731.1	244189.5	931.0	D	
55.11	92	16.16	85332.3	76799.1	432471.7	10662.4	421809.3	931.4	64405.3	6915.2
27.56	138	17.90	46333.2	41699.9	178807.1	13000.2	165806.9	2337.7	D	
41.33	138	17.26	68499.0	61649.1	267555.6	13931.2	253624.4	931.0	D	
**55.11**	**138**	**16.80**	**89999.0**	**80999.1**	**455384.3**	**14862.5**	**440521.7**	**931.4**	**18712.4**	**2009.2**
27.56	184	18.74	48999.8	44099.8	188504.3	17200.2	171304.1	2337.7	D	
41.33	184	17.51	66499.0	59849.1	261986.9	18131.2	243855.6	931.0	D	
55.11	184	17.35	88999.0	80099.1	457049.3	19062.6	437986.7	931.4	D	

Where: Bio = Biomass yield in dry bases, GCNP = Green cob number per hectare, GBN = Gross field benefit, TVC = Total variable cost, NB=Net benefit, MC = Marginal cost, MNB = Marginal net benefit, MRR = Marginal return, ETB = Ethiopian birr.

**Table 7:** Dominance and marginal rate of return analysis for the effect of population and nitrogen rate on grain- and straw yield of maize, combined over years (2021 and 2022).

Density in kg ha^−1^	N rate kg ha^−1^	Bio t ha^−1^	Gy kg ha^−1^	Adjusted Gy kg ha^−1^	GBN ETB ha^−1^	TVC ETB ha^−1^	NB ETB ha^−1^	MC	MNB	MRR
27.56	0	16.4	4572.4	4115.2	123429.9	0.0	123429.9			
41.33	0	15.8	5694.6	5125.2	136775.3	931.0	135844.4	931.0	12414.4	1333.5
55.11	0	15.4	5847.1	5262.4	149438.7	1862.3	147576.4	931.4	11732.0	1259.7
27.56	46	17.4	7359.1	6623.2	185078.1	4600.1	180478	2737.7	32901.6	1201.8
41.33	46	15.8	7947.0	7152.3	195408.9	5531.0	189877.9	931.0	9399.8	1009.7
55.11	46	15.7	8737.3	7863.5	212259.7	6462.4	205797.3	931.4	15919.4	1709.3
27.56	92	17.6	7225.0	6502.5	182401.4	8800.1	173601.2	2337.7	D	
41.33	92	16.9	7983.0	7184.7	197807.3	9731.1	188076.2	931.0	D	
**55.11**	**92**	**16.2**	**9682.9**	**8714.6**	**233388.5**	**10662.4**	**222726**	**931.4**	**16928.8**	**1817.6**
27.56	138	17.9	6345.7	5711.1	163921.1	13000.2	150920.9	2337.7	D	
41.33	138	17.3	8175.4	7357.8	202479.6	13931.2	188548.5	931.0	D	
55.11	138	16.8	8891.3	8002.1	217245.7	14862.5	202383.2	931.4	D	
27.56	184	18.7	6818.5	6136.7	175382.9	17200.2	158182.7	2337.7	D	
41.33	184	17.5	7646.8	6882.1	191427.9	18131.2	173296.7	931.0	D	
55.11	184	17.4	8170.7	7353.6	202513	19062.6	183450.4	931.4	D	

Where: Bio = Biomass yield in dry bases, Gy = Grain yield, GBN = Gross field benefit, TVC = Total variable cost, NB=Net benefit, MC = Marginal cost, MNB = Marginal net benefit, MRR = Marginal return, ETB = Ethiopian birr.

### 3.5 Correlation analysis

Grain yield was positively and significantly associated with Number of cob per plant, Cob diameter, Cob length, Biomass, Strawyield, (Table 8).

**Table 8:** Correlation analysis among growth, yield and yield components of food barley as influenced by N rate and variety.

	DT	MD	PHT	NCPP	CD	CL	BY	SY	GY	HI
**DT**	1									
**MD**	0.432**	1								
**PHT**	0.119^ns^	−0.013^ns^	1							
**NCPP**	0.075^ns^	0.194*	0.222*	1						
**CD**	0.070^ns^	0.087^ns^	−0.140^ns^	0.273*	1					
**CL**	0.148*	0.236^ns^	0.067^ns^	0.370**	0.786**	1				
**BY**	0.003^ns^	−0.062^ns^	0.072^ns^	0.201*	0.554**	0.461**	1			
**SY**	0.008^ns^	−0.091^ns^	0.092^ns^	0.177*	0.487**	0.397**	0.987**	1		
**GY**	0.021^ns^	0.009^ns^	0.036^ns^	0.250*	0.654**	0.580**	0.938**	0.879**	1	
**HI**	0.066^ns^	0.223*	−0.097^ns^	0.048^ns^	−0.051^ns^	0.0714^ns^	−0.639**	−0.724**	−0.368**	1

Where, DF = Degree of freedom, DT = Days to tasseling, DM = Days to maturity, PHT=Plant height, NCPP=Number of cob per plant, CD=Cob diameter, CL=Cob length, BIO=Biomass, SY=Straw yield, GY = Grain yield, HI=Harvest index, HSW=Hundred seed weight, and CV = coefficient of variation.

**Fig. 3:**
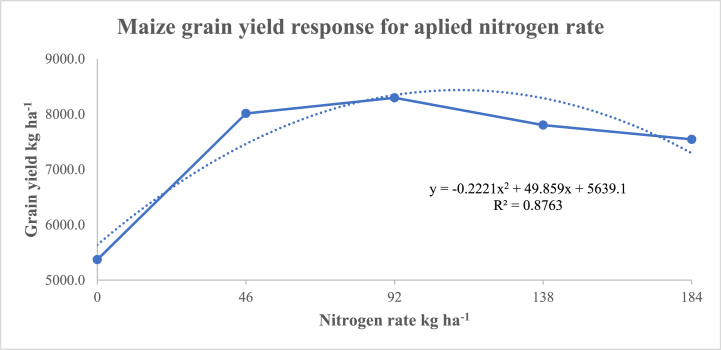
Relationship between grain yield of maize and nitrogen rate.

Stepwise regression analysis revealed that 88 % (R^2^ = 0.88) of the total variation of grain yield of maize was significantly explained by quadratic regression equation within the nitrogen rate range of 92–138 kg ha^−1^ for grain yield and green cob production respectively (Fig. 3).

## 4 Conclusion

The grain yield of maize increases with increasing plant population and nitrogen fertilizer level up to optimum. Maximum grain yield was obtained at the plant density of 88,888 plants ha^−1^ and from the tested nitrogen rate, the maximum grain yield was obtained from the rate of 92 kg ha^−1^ nitrogen, but statically similar grain yield was produced from 46 to 138 kg ha^−1^ nitrogen. Compared to the standard control of 44,444 plants ha^−1^ with the application of 46 kg N ha^−1^, the mean grain yield was increased by 24 % when the maize crop was sown at 88,888 plants ha^−1^ with the application of 92 kg N ha^−1^. Similarly, the green cob of maize increases with increasing the plant population, and nitrogen levels up to optimum. Maximum net benefit was obtained at the plant density of 88,888 plants ha^−1^ with the application of 138 kg N ha^−1^. Therefore, we recommend economically feasible treatment, which is 88,888 plants per hectare with the application of 92 kg N ha^−1^ best for the production of maize grain and 88,888 plant population with 138 kg N ha^−1^ for maize green cob production for all maize producers in the study and similar agro-ecological areas.

## Funding

This work was supported by the Debre Birhan 10.13039/501100024856Agricultural Research Center.

## Data availability

The data used to support the findings of this study are available from the corresponding author upon request.

## CRediT authorship contribution statement

**Degu Temeche:** Writing – original draft. **Elias Getachew:** Data curation. **Getachew Hailu:** Data curation.

## Declaration of competing interest

The authors declare that they have no known competing financial interests or personal relationships that could have appeared to influence the work reported in this paper.
